# Effects of Higher Serum Lipid Levels on the Risk of Parkinson's Disease: A Systematic Review and Meta-Analysis

**DOI:** 10.3389/fneur.2020.00597

**Published:** 2020-06-26

**Authors:** Zheng Jiang, Xinran Xu, Xiaojing Gu, Ruwei Ou, Xiaoyue Luo, Huifang Shang, Wei Song

**Affiliations:** Department of Neurology, West China Hospital, Sichuan University, Chengdu, China

**Keywords:** Parkinson's disease, cholesterol, triglyceride, systematic review, meta-analysis

## Abstract

**Background:** The causal relationship between serum lipid levels and the risk of Parkinson's disease (PD) remains largely uncertain. We summarized the existing controversial evidence on this topic.

**Methods:** We searched the electronic databases for observational studies from January 1988 to March 2020. We applied random-effects models to calculate pooled relative risk (RR) with their 95% confidence intervals (CI). Random-effects dose-response meta-analyses were further conducted to explore the dose-risk relationship.

**Results:** Twelve cohort studies and three case-control studies were included in this meta-analysis. Higher levels of serum low-density lipoprotein cholesterol (LDL-C) were inversely associated with the subsequent risk of PD (RR 0.73, 95% CI 0.57–0.93), whereas, there were no associations between serum levels of total cholesterol (TC) (RR 0.91, 95% CI 0.73–1.13), high-density lipoprotein cholesterol (HDL-C) (RR 0.97, 95% CI 0.73–1.27), or triglycerides (TG) (RR 0.85, 95% CI 0.55–1.29) and the risk of PD. Further dose-response meta-analysis revealed that every 38.6 mg/dL (1mmol/L) increase in serum LDL-C correlates with a 7% decreased risk of PD.

**Conclusions:** Our paper supports the protective effect of higher serum LDL-C on the subsequent risk of PD. More prospective cohort studies are warranted to confirm the conclusion, and further fundamental researches are needed to elucidate the underlying biological mechanisms.

## Introduction

Parkinson's disease (PD) is a severe neurodegenerative disease, affecting 1% of the population above the age of 60 (1). While familial forms of PD represent 5–10% of total cases (2), the majority of PD patients are sporadic with unknown causes. Previous studies have found that lipids are involved in most classical PD-related processes, such as oxidative stress. Thus altered lipid metabolism may play an essential role in the pathogenesis of PD (3–5).

Clinically, disrupted lipid metabolism can lead to abnormal serum lipid levels of TC, LDL-C, HDL-C, and TG. However, the causal relationship between serum lipid levels and the risk of PD has not yet been determined. Since the first attempt in 1994 (6), many epidemiological studies have reported on this association, but results are inconsistent. For example, several studies have found a lower risk of PD with higher serum TC (7–9). Conversely, another two studies suggested a positive causal association (10, 11), while more studies failed to find a significant linkage (12–17). Similarly, studies on other serum lipid parameters, including LDL-C, HDL-C, and TG, have yielded controversial conclusions.

Although a meta-analysis in 2013 observed no association between serum cholesterol and the risk of PD (18), more rigorous studies are emerging to support an inverse causal association of them, by adopting a more robust study design and adjusting more crucial confounders (8, 13, 19). Furthermore, as a vital parameter of serum lipids, the relationship of serum TG and PD risk has never been debated in any quantitative review. In this paper, we performed random-effects meta-analyses to synthesize the published evidence linking serum lipids to the risk of PD, and dose-response meta-analyses were also conducted to explore the dose-risk relationship with available data for serum TC and LDL-C.

## Methods

### Literature Search

Our meta-analysis followed the Preferred Reporting Items for Systematic Reviews and Meta-analyses (PRISMA) statement (see Supplementary PRISMA 2009 Checklist) (20). The authors (ZJ and XRX) independently searched MEDLINE, EMBASE, ACP Journal Club, COCHRANE, and so on via OvidSP. Titles and abstracts in duplicate were subsequently browsed to screen studies satisfying retrieval strategies from January 1988 to March 2020 without language limitations (see Supplementary Table 1). We then retrieved all relevant articles based on consensus among authors. Bibliographies of included articles were checked.

### Study Eligibility

Studies were included if they fulfilled the following criteria: (1) cohort or case-control study focusing on the association between serum lipid parameters (TC, LDL-C, HDL-C, and TG) and PD risk, (2) follow-up time>one year, and (3) providing information (blood samples or medical records or history of lipid-lowering agents) on serum lipid levels before PD onset. Exclusion criteria were as follows: (1) participants diagnosed as secondary parkinsonism or parkinsonism plus syndrome, or (2) studies lacking in risk estimates or without sufficient data to calculate them.

### Data Extraction and Quality Assessment

Two authors (ZJ and XRX) independently extracted the following data from included studies: first author and publication year; study location; study design; cohort name; serum lipid parameters; data type; follow-up time; number of participants/person-years, number of cases(overall and for each category); age; outcome definition (relative risk[RR]/odds ratio[OR]/hazard ratio[HR]); covariates adjusted; levels of exposure, along with corresponding risk estimates and 95% CI for each category. Disagreements were settled by mutual discussion. If articles selected in this paper report concentrations of serum lipids by the International System of Units (SI), we converted those to conventional units with a conversion ratio of 38.6 (1 mg/dL = 0.0259 mmol/L) for TC, LDL-C, and HDL-C or 88.5 (1 mg/dL = 0.0113 mmol/L) for TG.

The Newcastle-Ottawa Scale (NOS) was used to evaluate the quality of the included observational studies (21). A rating of ≥seven stars was deemed of high quality, and < seven stars were of poor quality. Any discrepancy was resolved by involving an arbiter (RWO).

### Statistical Analysis

Because PD is a relatively uncommon disease, HR or OR is considered to mathematically approximate RR (22). For high vs. low category meta-analysis, the DerSimonian and Laird random-effects models were executed to calculate pooled RRs and their 95% CIs (23). When some articles only provided divided RRs by gender for males and females, gender-specific RRs were combined by carrying out fixed-effects models for further analysis. Heterogeneity was assessed by Cochrane's Q test and *I*^2^ statistics, and a value of *I*^2^ above 50% or *P* < 0.1 indicated substantial heterogeneity (24). We performed subgroup analyses to search for the potential sources of heterogeneity. Publication bias was also explored by the funnel plot for at least ten available studies, together with Egger's test and Begg's test (*P* < 0.05). Sensitivity analysis was performed by removing one study at a time or switching analysis model to check the robustness of the results (25).

A dose-response meta-analysis was also performed to explore the dose-risk relationship between exposure and risk. The category serum lipid levels were determined as the mean or median value in each category if available for each study. For studies only gave the range in a category, the midpoint value was assigned for closed categories. In the case of open-ended highest or lowest category, the category serum lipid levels were equal to the lower or upper boundary plus or minus 1.5 folds the range of the closest category. When studies set the highest categories as the reference groups, we uniformly converted them to the lowest categories as needed (26). When the included studies just reported the number of total cases and overall person-years/participants, the distribution of cases and person-years/participants for each category was calculated using the methods proposed by Aune et al. (27)and Bekkering et al. (28). If researches only provided the number of cases and participants for each category, we could prudently estimate RRs and their 95% CIs using the methods raised by Bekkering et al. (28) and Orsini et al. (29) when necessary. We performed the random-effects dose-response meta-analysis by two steps (30–32). Step one: a restricted cubic spline model with three knots at the 25, 50, and 75% percentiles of the interval of exposure was estimated, and the two regression coefficients (three knots minus one) were calculated. Step two: we utilized a multivariate random-effects model to pool the variance/covariance matrix for each research. Non-linearity test was done by testing the null hypothesis, where the regression coefficient of the second spline equals zero.

All statistical analyses were performed using the software of Stata 12.0 (StataCorp, Texas, USA).

## Results

### Study Selection and Characteristics

**Figure 1:**
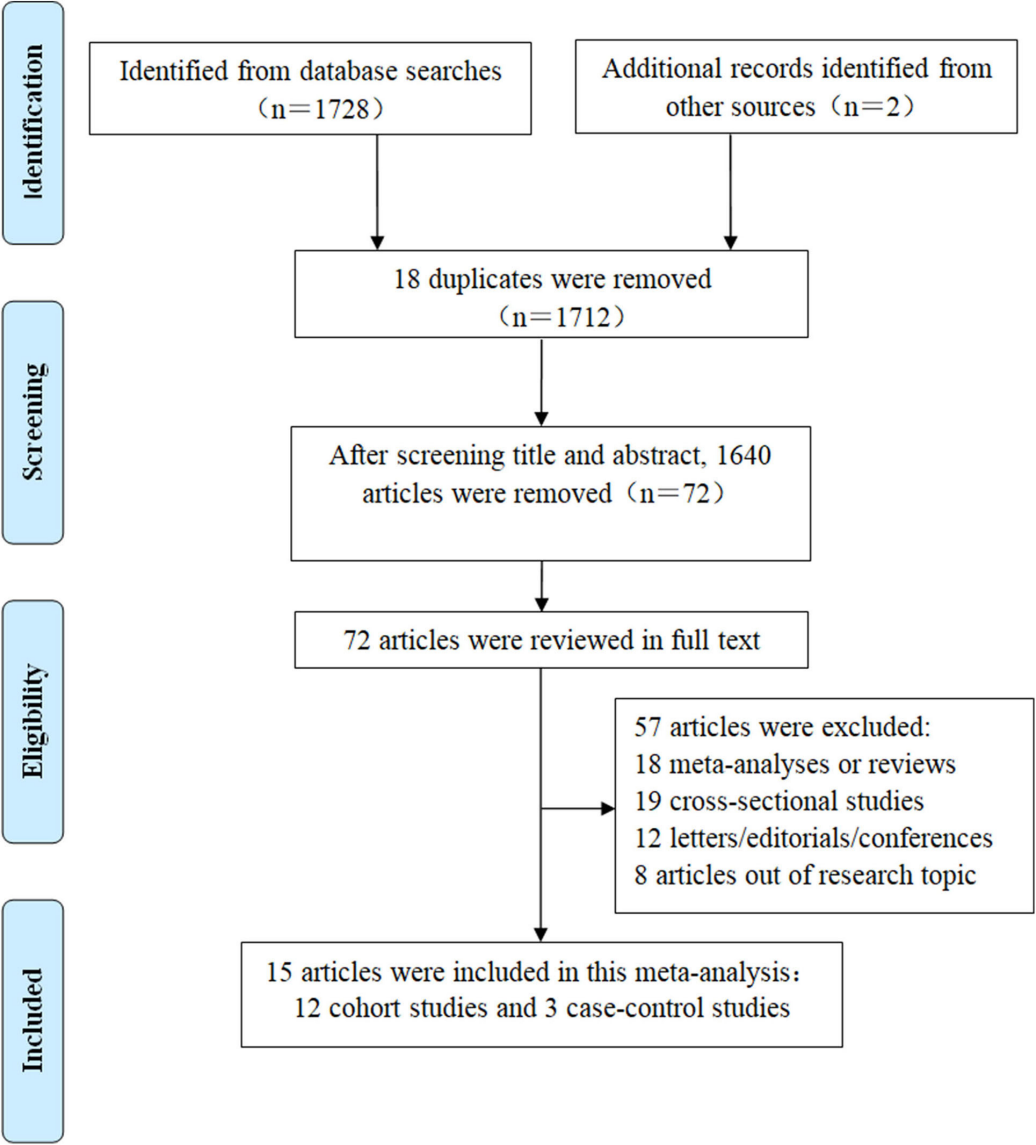
The flow chart for detailed steps of the literature search.

Detailed information on the literature search could be seen from Figure 1. We obtained 1,728 articles from the electronic databases. Another two records were acquired by viewing the references of related reviews or meta-analyses. After the exclusion of 18 repeats, we began screening the titles and abstracts of the rest 1,712 papers and therefore excluded 1,640 items. The full text of the remaining 72 articles was read, leading to the inclusion of 15 articles for the high vs. low analysis (6–10, 12–15, 19, 33–37) and nine articles for the dose-response analysis (8–10, 12, 14, 19, 33–35).

These 15 studies were published between 1994 and 2019. Three of the studies were performed primarily in Americas (9, 12, 15), five in Asia (6, 7, 19, 33, 36), and seven in Europe (8, 10, 13–15, 34, 35). A total of 49,956 cases were included. Thirteen studies included a mixed-gender population of males and females (7–10, 12–15, 19, 34–37); two studies only included males (6, 33). NOS scores ranged from five to nine (Table 1). We summarized the characteristics of the included studies in Supplementary Table 2A (for cohort studies) and Supplementary Table 2B (for case-control studies).

**Table 1:** Quality assessment of each eligible study according to the NOS.

**Study**	**Selection**				**Comparability**	**Outcome**		
	**1**	**2**	**3**	**4**	**1**	**1**	**2**	**3**
Rozani (2018)	*	*	*	*	**	*	*	*
Nam (2018)	*	*	*	*	*	*	*	*
Huang (2015)	*	*	*		**	*	*	*
Hu (2008)	*	*	*	*	*	*	*	*
Friedman (2013)	*	*	*	*	**	*		*
Simon (2007)		*		*	*	*	*	*
Saaksjarvi (2015)	*	*	*	*	*	*	*	*
Huang (2008)		*	*	*	*	*	*	*
Grandinetti (1994)		*	*	*		*	*	*
De Lau (2006)	*	*	*	*	*	*	*	*
Benn (2017)	*	*	*	*	*	*	*	*
Jeong (2019)	*	*	*	*	**	*	*	*
Vikdahl (2015)	*	*	*	*	*	*	*	*
Miyake (2010)	*	*		*	*		*	
Savica (2012)	*	*	*	*	*	*	*	

*NOS, Newcastle-Ottawa Quality Assessment Scale*.

### Data Synthesis

#### Serum TC and Risk of PD

##### High vs. low category meta-analysis

**Figure 2:**
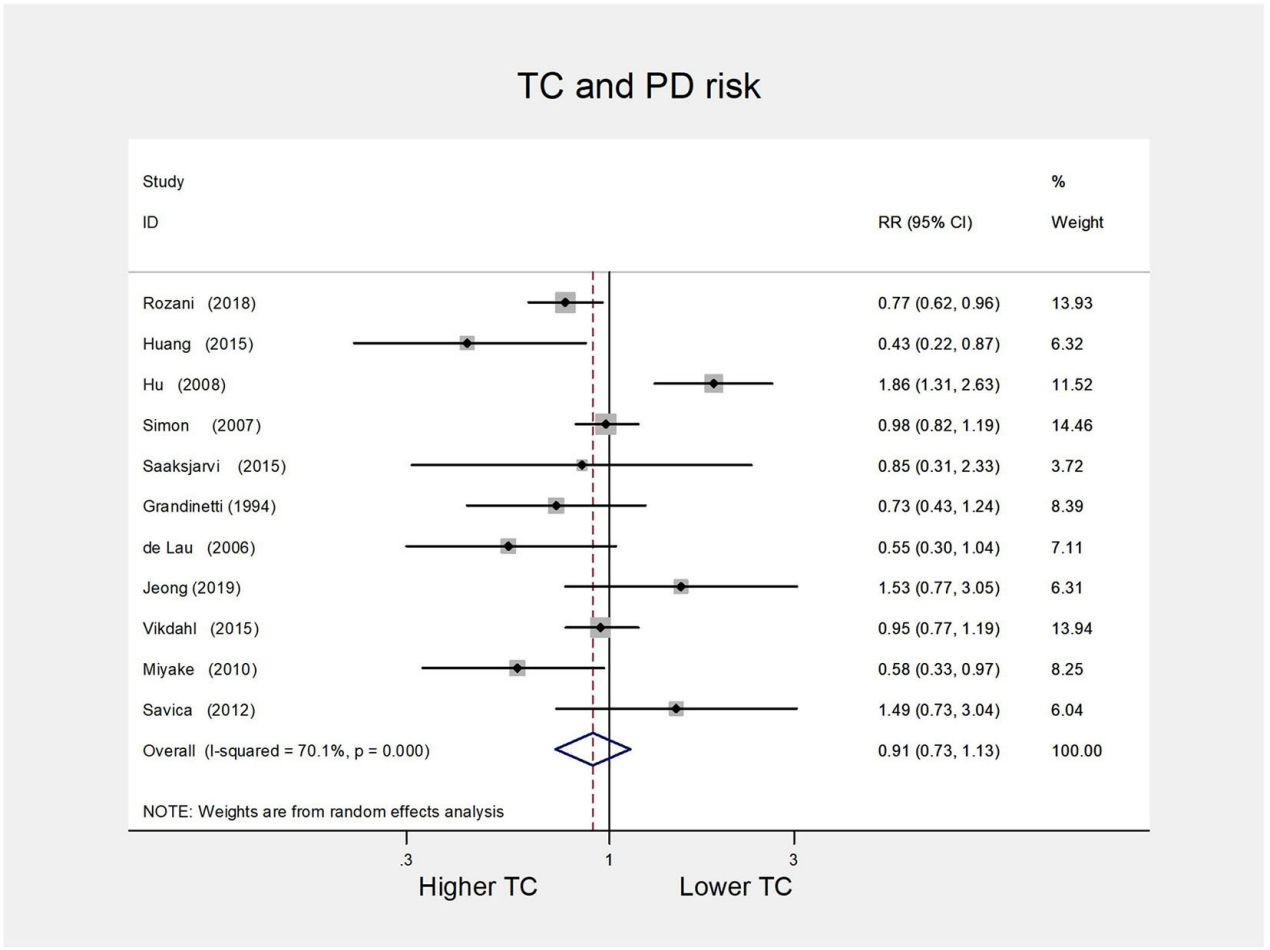
Forest plot presenting the pooled estimate effects (RR) on the relationship between serum TC levels and risk of PD. RR, relative risk; CI, confidence interval; TC, total cholesterol; PD, Parkinson's disease.

For the TC group, eight cohort studies (6, 8–10, 12–14, 19) and three case-control studies (7, 15, 37) were included for the high vs. low analysis involving 4,215 cases. We failed to discover a significant association between serum TC and the risk of PD (RR 0.91, 95% CI 0.73–1.13; *I*^2^= 70.1%, *P* = 0.000) (Figure 2).

##### Subgroup analysis

In subgroup analyses by study location, study design, statin use, quality of study, and gender, overall pooled risk estimate remained non-significant, and no evidence of between-study heterogeneity was found (*P* interaction > 0.05) (Table 2).

**Table 2:** Subgroup analyses (serum TC levels and PD risk).

**Variables**	**No. of studies**	**Test of association**		**Test of heterogeneity**		***P* interaction**
		**Pooled RR (95%CI)**	***P* value**	**I^**2**^ (%)**	***P* value**	
**Study location**						
Europe	5	0.95(0.66–1.37)	0.794	80.8	0.000	0.690
Americas	3	0.87(0.50–1.52)	0.631	70.3	0.034	
Asia	3	0.83(0.49–1.41)	0.492	59.1	0.087	
**Study design**						
cohort	8	0.90(0.67–1.21)	0.485	75.7	0.000	0.973
case-control	3	0.91(0.60–1.37)	0.635	57.0	0.098	
**Statin adjustment/excluding statin users**						
YES	6	1.01(0.65–1.57)	0.970	81.6	0.000	0.374
NO	5	0.87(0.71–1.05)	0.151	33.1	0.201	
**NOS**						
≥7	8	0.96(0.70–1.32)	0.810	76.1	0.000	0.467
<7	3	0.81(0.59–1.12)	0.201	49.4	0.139	
**Gender**						
Male	7	0.87(0.63–1.21)	0.414	66.8	0.006	0.918
Female	6	0.90(0.56–1.43)	0.649	71.3	0.004	

*RR, relative risk; CI, confidence interval; PD, Parkinson's disease; TC, total cholesterol; NOS, Newcastle-Ottawa Quality Assessment Scale. P value for heterogeneity within subgroup. P interaction for heterogeneity between subgroups with meta-regression analysis (at least ten studies)*.

##### Publication bias and sensitivity analysis

**Figure 3:**
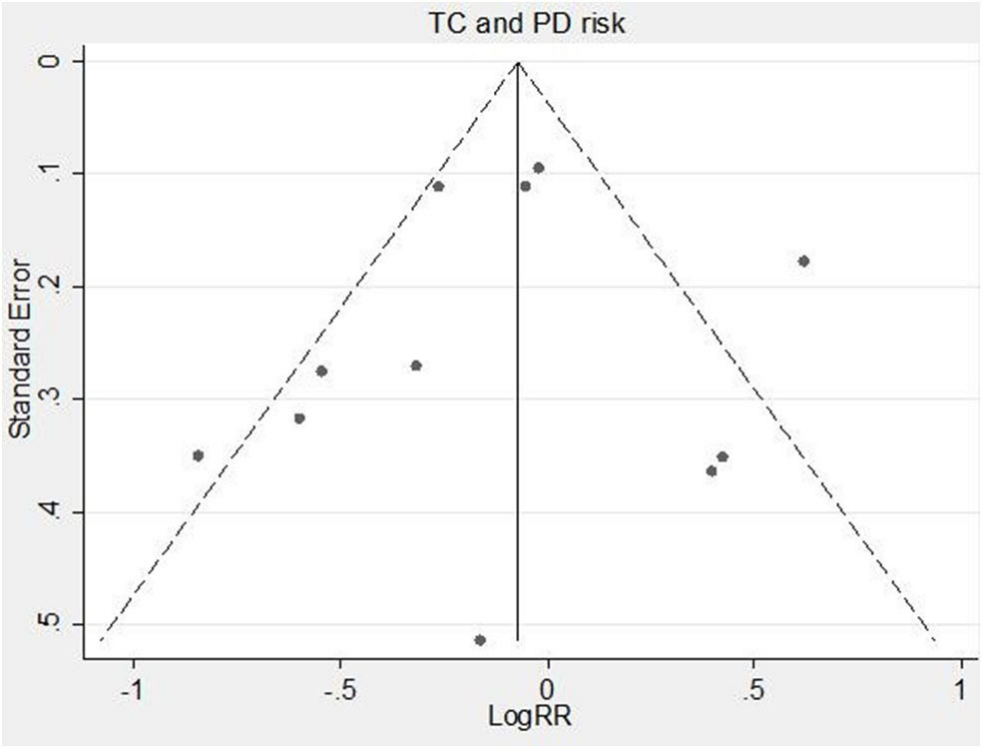
Funnel plot of serum TC levels and risk of PD. RR, relative risk; TC, total cholesterol; PD, Parkinson's disease.

The funnel plot was visually asymmetry (Figure 3). However, neither Egger's test (*P* = 0.749) nor Begg's test (*P* = 0.876) detected publication bias. Sensitivity analysis results are in the range from 0.84(95% CI 0.70–1.00) to 0.96(95% CI 0.77–1.19) by deleting one related study each time, suggesting the stability of our meta-analysis.

##### Dose-response meta-analysis

**Figure 4:**
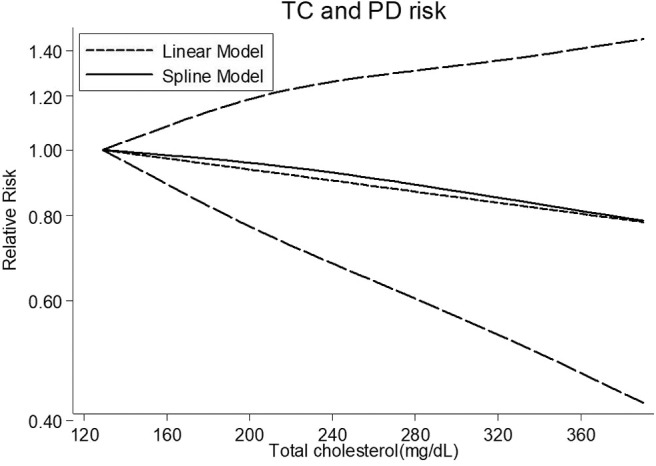
Dose-response relationship between serum TC levels and risk of PD. TC, total cholesterol; PD, Parkinson's disease.

Five cohort studies (8, 10, 12, 14, 19) were chosen for the dose-response analysis between serum TC and the risk of PD, involving 3,433 cases (Supplementary Table 3). Results revealed that the test for nonlinearity was not significant (*P* = 0.732), and there was no linear dose-risk relationship between them (*P* = 0.413) with substantial heterogeneity (*P* = 0.000) (Figure 4).

#### Serum LDL-C and Risk of PD

##### High vs. low category meta-analysis

**Figure 5:**
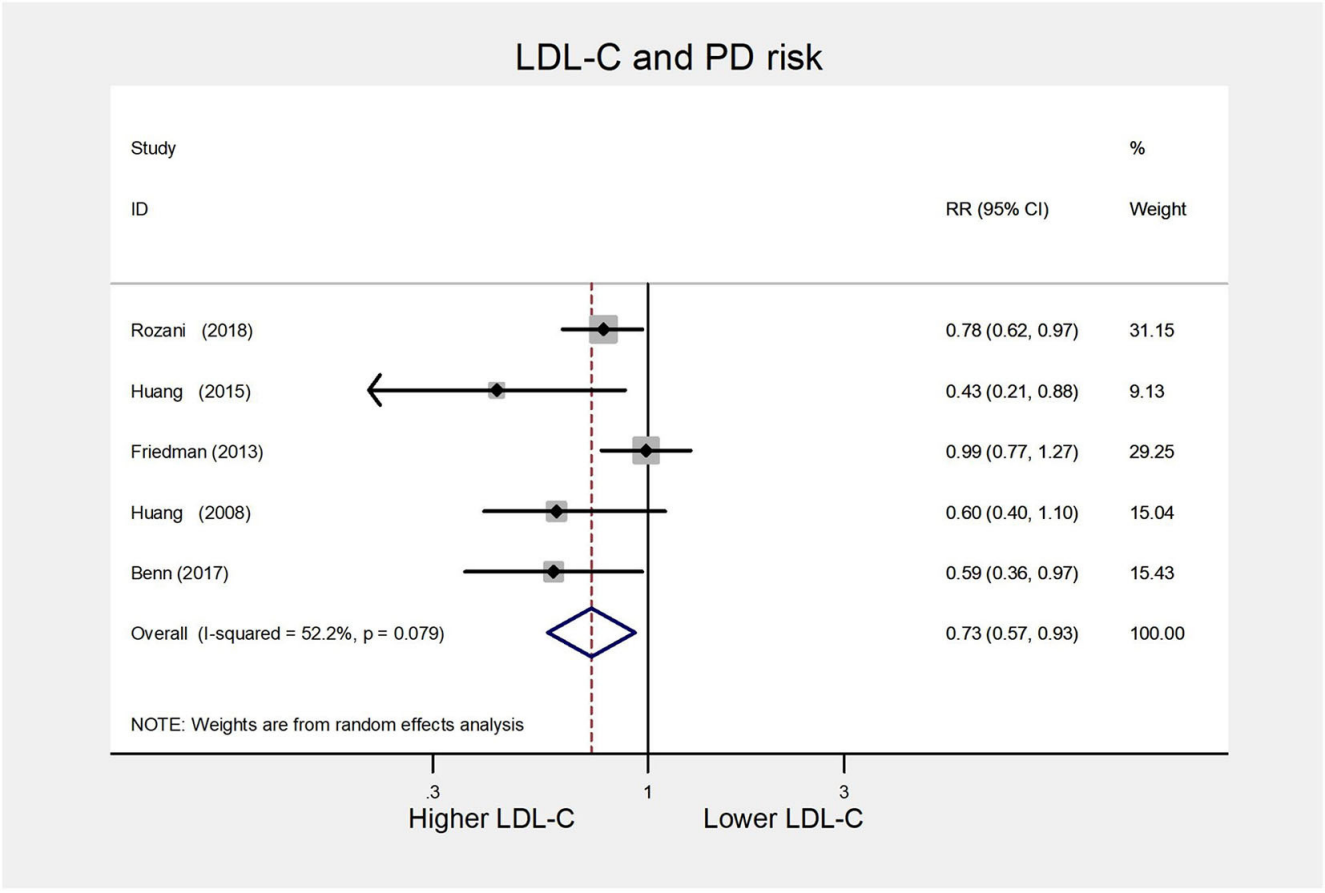
Forest plot presenting the pooled estimate effects(RR) on the relationship between serum LDL-C levels and risk of PD. RR, relative risk; CI, confidence interval; LDL-C, low-density lipoprotein cholesterol; PD, Parkinson's disease.

For the LDL-C group, five cohort studies (8, 9, 33–35) were included for the high vs. low analysis involving 2,406 cases. All five studies were of high quality (NOS scores ≥ 7). Our results implied that higher serum LDL-C could reduce the subsequent risk of PD (RR 0.73, 95% CI 0.57–0.93; *I*^2^= 52.2%, *P* = 0.079) (Figure 5).

##### Subgroup analysis

In subgroup analyses according to study location, statin use, and gender, similar significant inverse relationships between serum LDL-C and PD risk were observed for studies confined to a location in Americas (RR 0.43, 95% CI 0.21–0.88), statin adjustment (RR 0.66, 95% CI 0.49–0.90), or male (RR 0.69, 95% CI 0.54–0.88). Besides, the substantial heterogeneity disappeared in the male group. We summarized the detailed results in Table 3.

**Table 3:** Subgroup analyses (serum LDL-C levels and PD risk).

**Variables**	**No. of studies**	**Test of association**		**Test of heterogeneity**	
		**Pooled RR (95%CI)**	***P* value**	**I^**2**^ (%)**	***P* value**
**Study location**					
Europe	3	0.82 (0.64–1.04)	0.099	50.3	0.134
Americas	1	0.43 (0.21–0.88)	0.021	—	—
Asia	1	0.60 (0.40–1.10)	0.099	—	—
**Statin adjustment/excluding statin users**					
YES	3	0.66 (0.49–0.90)	0.006	35.3	0.213
NO	2	0.81 (0.50–1.31)	0.085	66.9	0.082
**Gender**					
Male	2	0.69 (0.54–0.88)	0.003	0	0.537
Female	1	0.88 (0.62–1.29)	0.494	—	—

*RR, relative risk; CI, confidence interval; NOS, Newcastle-Ottawa Scale; PD, Parkinson's disease; LDL-C, low-density lipoprotein cholesterol. P value for heterogeneity within subgroup*.

##### Publication bias and sensitivity analysis

Both Egger's test (*P* = 0.081) and Begg's test (*P* = 0.221) did not detect publication bias. Sensitivity analysis results were in the range from 0.67(95% CI 0.46–0.99) to 0.78(95% CI 0.62–0.98), suggesting that exclusion of any study did not substantially affect the pooled results. We also found a similar pooled result (RR 0.79 95% CI 0.68–0.91; *I*^2^= 52.2%, *P* = 0.079) using a fixed-effects model.

##### Dose-response meta-analysis

**Figure 6:**
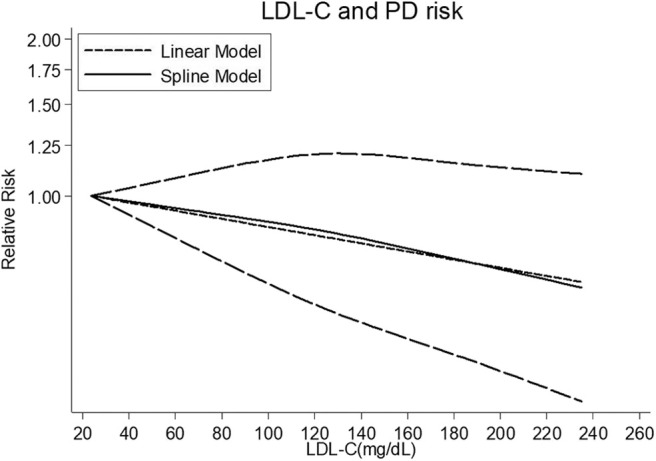
Dose-response relationship between serum LDL-C levels and risk of PD. LDL-C, low-density lipoprotein cholesterol; PD, Parkinson's disease.

Four cohort studies (8, 33–35) were included for the dose-response analysis, involving 2,300 cases (Supplementary Table 4). There was no sign of departure from linearity (*P* = 0.666). The RR of PD per 38.6 mg/dL (1mmol/L) increase in serum LDL-C concentration was 0.93 (95% CI 0.88–0.99), with little heterogeneity in the dose-response analysis (*P* = 0.084) (Figure 6).

#### Serum HDL-C and Risk of PD

##### High vs. low category meta-analysis

**Figure 7:**
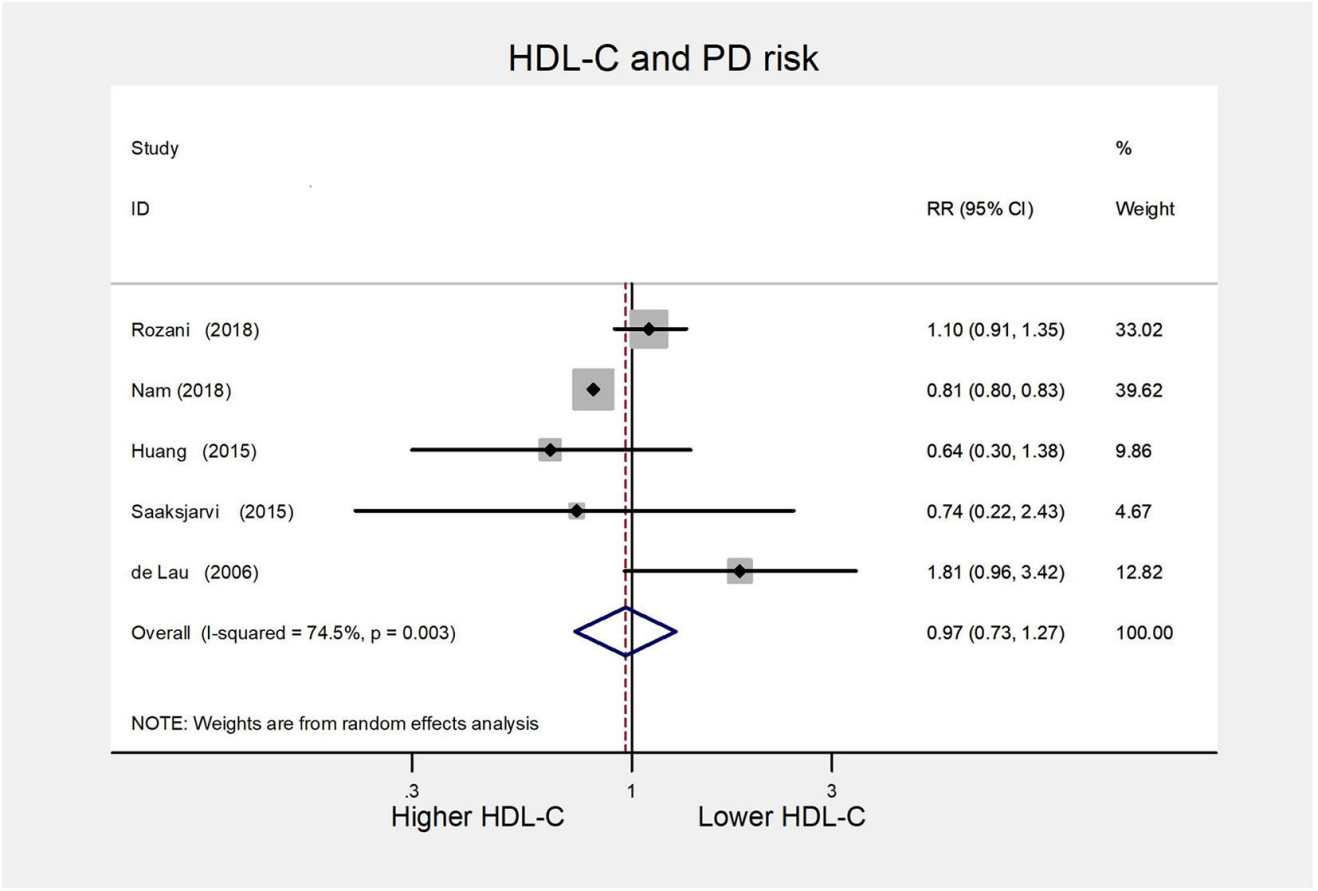
Forest plot presenting the pooled estimate effects(RR) on the relationship between serum HDL-C levels and risk of PD. RR, relative risk; CI, confidence interval; HDL-C, high-density lipoprotein cholesterol; PD, Parkinson's disease.

For the HDL-C group, we included five cohort studies (8, 9, 13, 14, 36) for the high vs. low analysis involving 45,251 cases. The summary RR was 0.97(95% CI 0.73–1.27; *I*^2^= 74.5%, *P* = 0.003) (Figure 7), suggesting the lack of association between serum HDL-L and PD risk.

##### Subgroup analysis

As all included studies were of high quality, we conducted subgroup analyses following study location, statin use, and gender. The pooled risk estimate remained non-significant, and between-study heterogeneity decreased in either Europe or the male group (Supplementary Table 5).

##### Publication bias and sensitivity analysis

There was no indication of publication bias assessed with Egger's test (*P* = 0.308) and Begg's test (*P* = 1.000). Sensitivity analysis results range from 0.89 (95% CI 0.70–1.13) to 1.08 (95% CI 0.76–1.55) by omitting one study in order, confirming that the original results were reliable.

#### Serum TG and Risk of PD

##### High vs. low category meta-analysis

**Figure 8:**
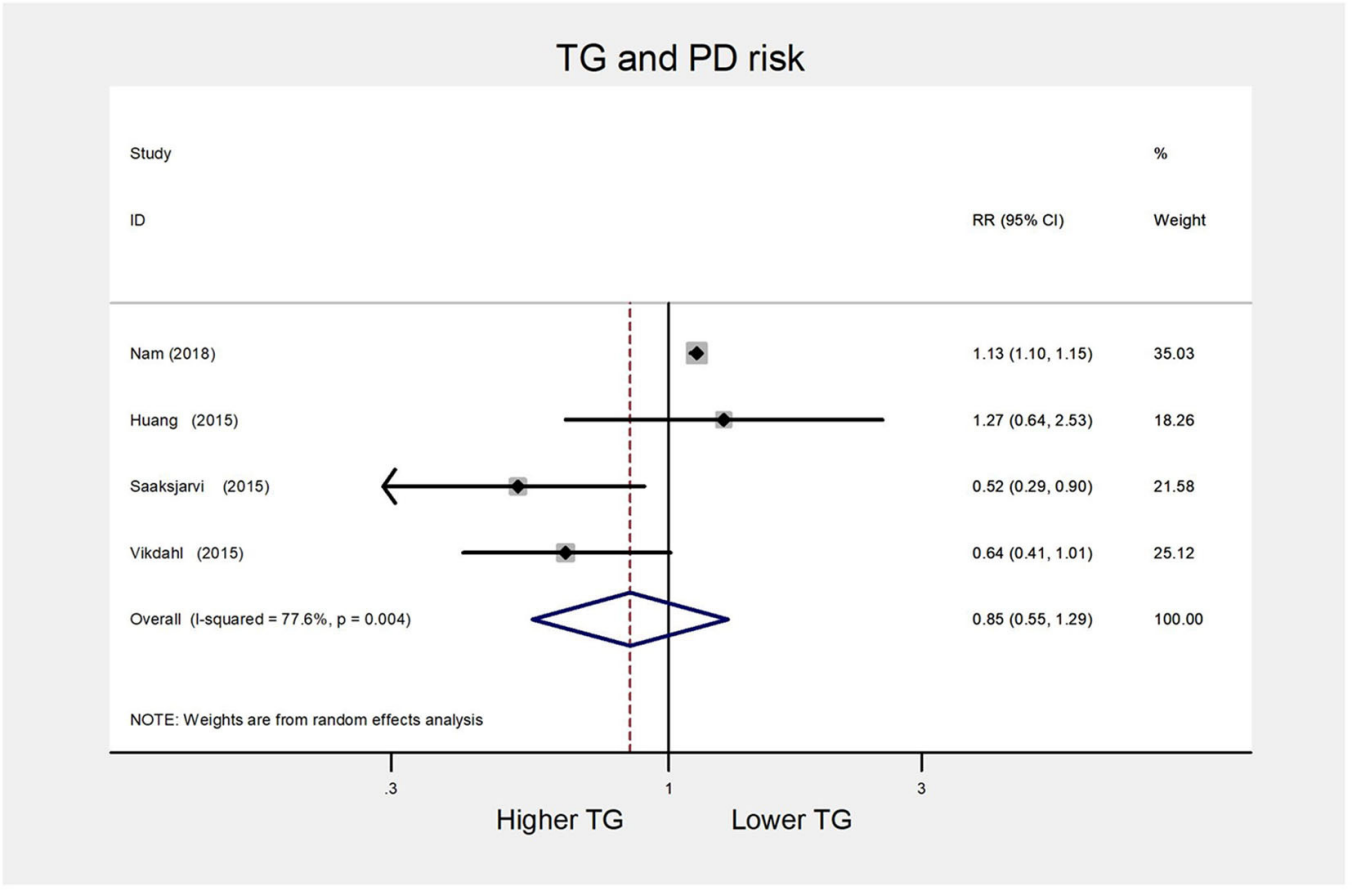
Forest plot presenting the pooled estimate effects(RR) on the relationship between serum TG levels and risk of PD. RR, relative risk; CI, confidence interval; TG, triglycerides; PD, Parkinson's disease.

For the TG group, we obtained three cohort studies (9, 13, 36) and one case-control study (15) for the high vs. low analysis involving 44,484 cases. As presented in Figure 8, the combined RR was 0.85 (95% CI 0.55–1.29) with evidence of substantial heterogeneity (*I*^2^= 77.6%, *P* = 0.004). Due to limited included studies, we could not perform subgroup analysis and evaluate publication bias.

##### Sensitivity analysis

Sensitivity analyses indicated little change in the pooled RR when any study was removed (data not shown).

## Discussion

In this meta-analysis, we summarized the current evidence on the association between serum lipid parameters and the risk of PD, and thus provides a unique perspective to evaluate the effects of different serum lipid parameters on PD risk. There are some notable strengths deserving mention. Firstly, four serum lipid parameters, including TC, LDL-C, HDL-C, and TG, were included in a quantitative review to investigate their effects on PD risk for the first time. Secondly, the strict inclusion criterion of providing information on serum lipid levels before PD onset avoided the possibility of reverse causality from abnormal lipid profile induced by PD. Thirdly, both the high vs. low analysis and dose-response analysis were conducted, which ensures the reliability of the outcome.

In the high vs. low analysis, we found that the exposure of higher serum LDL-C levels may decrease the future risk of PD, which is consistent with the previous data on the association between serum LDL-C and PD risk (8, 9, 11, 17, 35). There was also a significant inverse linear dose-risk relationship that each 38.6 mg/dL(1 mmol/L) increase in serum LDL-C was associated with a 7% relative reduction in PD risk. Meanwhile, our result is different from that of the earlier meta-analysis conducted by Sheng et al. (38). They systematically reviewed three cohort studies and found that the high baseline levels of serum LDL-C was not significantly associated with the risk of PD (RR 0.58, 95% CI 0.31–1.07; *P* = 0.080). However,the small number of included studies, as well as significant heterogeneity (*I*^2^= 71%) in their meta-analysis, indicated their result was unreliable and unstable. In addition, no significant association between serum TC and PD risk in the high vs. low analysis or dose-response analysis was observed, which was in line with the prior finding by Gudala et al. (18). For other serum lipid parameters of HDL-C and TG, we were unable to note a significant relationship between them and PD risk.

Multiple lines of biological evidence responsible for the inverse linkage between serum cholesterol and PD risk have been found. Some rodent experiments supported the protective role of cholesterol precursors in the pathogenesis of PD. For example, squalene might save the striatal neurons from toxic effects caused by 6-hydroxydopamine (39), and lanosterol could protect dopaminergic neurons in the substantia nigra from dying induced by 1-methyl-4-phenyl-1,2,3,6-tetrahydropyridine (40). Furthermore, high plasma cholesterol could be a marker of coenzyme Q10, a molecule serving as a crucial endogenous anti-oxidant as well as an essential electron acceptor of mitochondrial respiratory chain complexes I and II (41, 42). Its potential neuroprotective effects had been demonstrated *in vitro* studies and animal models of PD (43). Besides, lower serum cholesterol levels have been identified as potential markers of several prodromal symptoms of PD before diagnosis, such as constipation (44), olfactory dysfunction (45), depression, and reduced activity, which might affect serum cholesterol levels (46, 47).

In contrast, some hypotheses support a positive relationship between serum cholesterol levels and PD risk. As is generally recognized, abnormal intracellular aggregation of α-synuclein has an etiological role in the occurrence and development of PD (48). Elevated serum cholesterol could accelerate the pathological process of α-synuclein. Cholesterol seems to involve in α-synuclein accumulation (49, 50) and aggregation (51) and induces α-synuclein to form pores (52, 53). Moreover, some studies have provided evidence that overweight is associated with an increased PD risk (54–56). Another study suggested that body mass index positively correlated with serum cholesterol levels (10). So we suspect that high serum cholesterol might contribute to the occurrence of PD partly through excessive body weight.

While exploring serum lipids in relation to the risk of PD, we should draw more attention to the confounding effect of lipid-lowering drugs on this association. Statins are cholesterol-lowering drugs widely prescribed to prevent and treat cardiovascular and cerebrovascular diseases. Some studies indicated that statins might have anti-inflammation activity (57) and slow down the intraneuronal aggregation of α-synuclein *in vitro* and *in vivo* experiments (51, 58). Furthermore, two recent meta-analyses (38, 59) suggested that statin use was associated with reduced PD risk. Nonetheless, most of the studies examining the relationship between serum lipids and PD risk overlooked the potential confounding effect of statins. The above, coupled with the tendency to use statins for those with hypercholesterolemia, make it difficult to ascertain whether higher serum cholesterol is the protective factor of PD. Therefore, in the high vs. low analysis assessing serum LDL-C in relation to PD risk, subgroup analyses stratified by statin use were performed to exclude the potential confounding effect. The inverse association between serum LDL-C and PD risk remained significant with adjustment for statin use. However, the association became non-significant without adjustment for statin use, indicating that the confounding factor of statin use could possibly underestimate the protective effect of higher serum LDL-C on PD risk.

For this paper, we should consider several limitations. Firstly, as mentioned before, altered serum lipid levels might represent a prodromal symptom of PD. Without taking into consideration the duration of the prodromal period, while still challenging to ascertain, we include the studies using data of serum lipid levels measured just before the onset of PD, which might make the results somewhat biased. Secondly, a limited number of the included studies for the relationship between serum TG and PD risk urges us to interpret its negative results with caution. Thirdly, there are some missing data in the dose-response analysis, and the approximate values inferred by existing methods may not be accurate, so the results of the dose-risk relationship might demand more sufficient data.

## Conclusion

Our meta-analysis showed that higher serum LDL-C might be a protective factor for PD and somehow reduce the future risk of PD. From an overall perspective, the protective effect of high serum LDL-C on PD risk attends to trifles and neglects essentials. After all, the increasing threat of cardiovascular and cerebrovascular events induced by hypercholesterolemia is a major public health concern. However, this study still has some implications for exploring the role of cholesterol in the pathogenesis of PD.

## Data Availability Statement

All datasets generated for this study are included in the article/Supplementary Material.

## Author Contributions

ZJ, XX, and RO designed the study and did the literature review. ZJ did the statistical analysis, edited tables and pictures, and wrote the primary manuscript. XG contributed to revising the grammar. XL helped to promote the methodology. HS and WS reviewed the final manuscript. All authors contributed to the article and approved the submitted version.

## Conflict of Interest

The authors declare that the research was conducted in the absence of any commercial or financial relationships that could be construed as a potential conflict of interest.
