# Supplementary material for: Effects of Higher Serum Lipid Levels on the Risk of Parkinson's Disease: A Systematic Review and Meta-Analysis
Source: Front Neurol. 2020 Jun 26;11:597. doi: 10.3389/fneur.2020.00597 (PMC7332704; doi:10.3389/fneur.2020.00597)
Supplement: Supplementary file 1 [file Table_2.DOC]

**Supplementary Table 1** MEDLINE, EMBASE, ACP Journal Club, COCHRANE, and so on via OvidSP.

| 1. exp Parkinson Disease/ |
| --- |
| 2. exp parkinsonism/ |
| 3. Parkinson$.ab,ti. |
| 4. (PD or IPD).ab,ti. |
| 5. (paralysis adj2 agitans).ab,ti. |
| 6. or/1-5 |
| 7. exp cholesterol/ |
| 8. (cholesterol$ or epicholesterol$ or azacosterol$ or diazacholesterol$ or hydroxycholesterol$ or 19-iodocholesterol$ or iodocholesterol$ or ketocholesterol$ or oxocholesterol$ or HDL or LDL or HDL-C or LDL-C or lipoprotein$ or triglyceride$ or hypercholester$ or hyperlipoprotein$ or hyperlipidemi$ or hypertriglyceridemi$ or hypocholester$ or hypolipoprotein$ or hypolipidemi$ or hypotriglyceridemi$).ab,ti. |
| 9. or/7-8 |
| 10. 6 and 9 |
| 11. exp animals/ not humans.sh. |
| 12. 10 not 11 |
| 13. remove duplicates from 12 |
| 14. limit 13 to yr=”1988-current” |

Specific Electronic Databases with Dates of Coverage:

EBM Reviews - Cochrane Database of Systematic Reviews (2005 to March 19, 2020),

EBM Reviews - ACP Journal Club (1991 to February 2020),

EBM Reviews - Database of Abstracts of Reviews of Effects (1st Quarter 2016),

EBM Reviews - Cochrane Clinical Answers (March 2020),

EBM Reviews - Cochrane Central Register of Controlled Trials (February 2020),

EBM Reviews - Cochrane Methodology Register (3rd Quarter 2012),

EBM Reviews - Health Technology Assessment (4th Quarter 2016),

EBM Reviews - NHS Economic Evaluation Database (1st Quarter 2016),

Embase (1974 to 2020 March 25),

Ovid MEDLINE(R) and Epub Ahead of Print, In-Process & Other Non-Indexed Citations, Daily and Versions(R) (March 25, 2020)

**Supplementary Table 2A Characteristics of included cohort studies.**

| **Author (Year)** | **Study location** | **Cohort Name** | **Serum lipid parameters** | **Data** | **Follow-up time(years)** | **Cohort size (% M)** | **Age(years)** | **Cases** | **Outcome definition** | **Exposure** | **Quantity** | **RR(95% CI)** | **Variables adjusted** | **NOS** |
| --- | --- | --- | --- | --- | --- | --- | --- | --- | --- | --- | --- | --- | --- | --- |
| Rozani(2018)† | Israel/Europe | Maccabi Health Services | TC, LDL-C, HDL-C | Dose-response + High vs. Low | 7.9 (mean) | 261638  (42.7) | M 47.1(mean) F 45.6(mean) | 764 | HR | TC | M tertiles:3 vs. 1 F tertiles:3 vs. 1 | 0.71(0.55–0.93) 0.93(0.63–1.38) | age, sex | 9/9 |
|  |  |  |  |  |  |  |  |  |  | LDL-C | M tertiles:3 vs. 1 F tertiles:3 vs. 1 | 0.72(0.54–0.95) 0.88(0.62–1.29) |  |  |
|  |  |  |  |  |  |  |  |  |  | HDL-C | M tertiles:3 vs. 1 F tertiles:3 vs. 1 | 1.06(0.83–1.34) 1.21(0.85–1.74) |  |  |
| Nam(2018) | Korea/Asia | National Health Insurance Service of South Korea | HDL-C, TG | High vs. Low | 5.3 (mean) | 17163560  (48.8) | ≥40 | 44205 | HR | HDL-C (mg/dL) | M ≥40 vs. ＜40* F≥50 vs. ＜50* | 0.81(0.80–0.83) | age, sex, smoking, alcohol, physical activity, income, BMI, eGFR, history of stroke | 8/9 |
|  |  |  |  |  |  |  |  |  |  | TG (mg/dL) | ≥150 vs. ＜150 | 1.13(1.10–1.15) |  |  |
| Huang(2015) | US/Americas | Atherosclerosis Risk in Communities Study | TC, LDL-C, HDL-C, TG | Dose-response + High vs. Low | 21 | 15291  (44.9) | 54.2(mean) | 106 | OR | TC | Tertiles 3 vs. 1 | 0.43(0.22–0.87) | age, sex, race, smoking, caffeine intake, statin | 8/9 |
|  |  |  |  |  |  |  |  |  |  | LDL-C | Tertiles 3 vs. 1 | 0.43(0.21–0.88) |  |  |
|  |  |  |  |  |  |  |  |  |  | HDL-C | Tertiles 3 vs. 1 | 0.64(0.30–1.38) |  |  |
|  |  |  |  |  |  |  |  |  |  | TG | Tertiles 3 vs. 1 | 1.27(0.64–2.53) |  |  |
| Hu(2008) | Finland/Europe | Monitoring Trends and Determinants of Cardiovascular Disease protocol | TC | Dose-response + High vs. Low | 18.1 (mean) | 50926  (48.6) | 25–74 | 625 | HR | TC (mg/dL) | Both ≥270 vs. ＜193 M ≥270 vs. ＜193 F ≥270 vs. ＜193 | 1.86(1.31–2.63) 1.84(1.14–2.95) 1.86(1.11–3.13) | age, study year, BMI, SBP, education, leisure-time physical activity, smoking, alcohol, coffee, tea, cholesterol-lowering agent use, diabetes | 8/9 |
| Friedman(2013) | Israel/Europe | Health services clinical database | LDL-C | Dose-response + High vs. Low | 7 | 94308  (47.2) | ＞45 | 1035 | OR | LDL-C (mg/dL) | ＞160 vs. ＜100 | 0.99(0.77–1.27) | sex, low socioeconomic status, diabetes, ischemic heart disease, hypertension, cerebrovascular accidents, smoking, statin | 8/9 |
| Simon(2007) | US/Americas | Nurses' Health Study(NHS), Health Professionals Follow-up Study(HPFS) | TC | Dose-response + High vs. Low | NHS  12.6 (mean)  HPFS  18.1 (mean) | 171879  (29.6) | M 40–75 F 30–55 | 530 | RR | TC (high cholesterol) | Both yes vs. no M yes vs. no F yes vs. no | 0.98 (0.82–1.19) 0.36(0.17–0.80) 0.54 (0.22–1.30) | age, smoking | 6/9 |
| Saaksjarvi(2015) | Finland/Europe | Mini-Finland Health Survey | TC, HDL-C, TG | High vs. Low | 30 | 6641  (46.7) | 30–79 | 89 | RR | TC (mg/dL) | ≥193 vs. ＜193 | 0.85 (0.31–2.33) | age, sex, education, smoking, alcohol, exercise, serum vitamin D, coffee | 8/9 |
|  |  |  |  |  |  |  |  |  |  | HDL-C (mg/dL) | M ≥39 vs. ＜39 F ≥50 vs. ＜50 | 0.74(0.22–2.43) | age, sex, education, smoking, alcohol, exercise, serum vitamin D, coffee, BMI, blood pressure, TG or HDL-C, FBG |  |
|  |  |  |  |  |  |  |  |  |  | TG (mg/dL) | ≥150 vs. ＜150 | 0.52(0.29–0.90) |  |  |
| To be continued | | | | | | | | | | | | | | |
| Huang(2008) | Japan/Asia | Honolulu-Asia Aging Study | LDL-C | Dose-response + High vs. Low | 3.3 (mean) | 3233  (100) | 77(mean) | 41 | RR | LDL-C (mg/dL) | ≥135 vs. ＜85 | 0.60(0.40–1.10) | age, smoking, coffee intake, bowel movement frequency, HDL-C, alcohol intake, presence of Apo e2 alleles, CASI | 7/9 |
| Grandinetti(1994)† | Japan/Asia | Honolulu-Asia Aging Study | TC | High vs. Low | 26 | 8006  (100) | 71–93 | 58 | RR | TC (high cholesterol) | Yes vs. no | 0.73(0.43–1.24) | age | 6/9 |
| De Lau(2006) | Netherland/Europe | Rotterdam Study | TC, HDL-C | Dose-response + High vs. Low | 9.4 (mean) | 6465  (41.1) | 69(mean) | 87 | HR | TC (mg/dL) | Both ＞286 vs. ＜228 M ＞274 vs. ＜216 F ＞293 vs. ＜236 | 0.55(0.30–1.04) 0.86(0.36–2.02) 0.16(0.05–0.55) | age, sex | 8/9 |
|  |  |  |  |  |  |  |  |  |  | HDL-C (mg/dL) | Both ＞62 vs. ＜43 M ＞54 vs. ＜39 F ＞66 vs. ＜46 | 1.81(0.96–3.42) 1.38(0.56–3.43) 2.69(1.11–6.49) |  |  |
| Benn(2017) † | Denmark/Europe | Copenhagen General Population Study, Copenhagen City Heart Study | LDL-C | Dose-response + High vs. Low | 8.2 (median) | 111194  (45) | 56(median) | 460 | HR | LDL-C (mg/dL) | ≥154 vs. ＜70* | 0.59(0.36–0.97) | age, sex, birth year, smoking, alcohol, physical inactivity, income, education, menopause for women | 8/9 |
| Jeong(2019)† | Korea/Asia | National Health Screening Program | TC | Dose-response + High vs. Low | 9.9(median) | 76043  (51.5) | 68.5(mean) | 1427 | HR | TC (mg/dL) | ≥300 vs. 200–240 | 1.53(0.77–3.05) | age, sex, BMI, income, smoking, alcohol, physical activity, hypertension, diabetes, SBP, FBG | 9/9 |

*Reference values were converted to the lowest exposure category form(see method). †: excluding statin users in the high vs. low analysis for serum lipid parameters. OR: odds ratio, RR: relative risk, HR: hazard ratio, CI: confidence interval, TC: total cholesterol, LDL-C: low-density lipoprotein cholesterol, HDL-C: high-density lipoprotein cholesterol, TG: triglycerides, PD: Parkinson’s disease, NOS: Newcastle-Ottawa Scale, US: the United States of America. NA: not available, BMI: Body Mass Index, CASI: Cognitive Abilities Screening Instrument, SBP: systolic blood pressure, FBG: fasting blood glucose,eGFR: estimated glomerular filtration rate, M: Male, F: female.

| **Author (Year)** | **Study location** | **Serum lipid parameters** | **Data** | **Cases (% male)** | **Controls (% male)** | **Age** | **Outcome definition** | **Exposure** | **Quantity** | **RR(95% CI)** | **Variables adjusted** | **NOS** |
| --- | --- | --- | --- | --- | --- | --- | --- | --- | --- | --- | --- | --- |
| Vikdahl(2015) | Sweden/Europe | TC, TG | High vs. Low | 84(54.7) | 336(54.7) | NA | HR | TC (mg/dL) | Both Per 38.6mg/dL increase M Per 38.6mg/dL increase F Per 38.6mg/dL increase | 0.95(0.77–1.19) 0.91(0.68–1.23) 0.95(0.68–1.33) | age, BMI, physical activity, smoking | 8/9 |
|  |  |  |  |  |  |  |  | TG (mg/dL) | Per 88.5mg/dL increase | 0.64(0.41–1.01) |  |  |
| Miyake(2010) | Japan /Asia | TC | High vs. Low | 249(37.4) | 368(38.3) | Cases 68.5(mean) Controls 66.6(mean) | OR | TC (high cholesterol) | Yes vs. no | 0.58(0.33–0.97) | sex, age, region of residence, smoking, education, leisure-time exercise, BMI, dietary energy, cholesterol, vitamin E, alcohol, coffee, DGI | 5/9 |
| Savica(2012)† | US/Americas | TC | High vs. Low | 196(61.7) | 196(61.7) | Cases 71(median) Controls NA | OR | TC (mg/dL) | ＞300 vs. ＜300 | 1.49(0.73–3.04) | age, sex, smoking, coffee | 7/9 |

**Supplementary Table 2B** Characteristics of included case-control studies.

†: excluding statin users in the high vs. low meta-analysis for serum lipid parameters. OR: odds ratio, RR: relative risk, HR: hazard ratio, CI: confidence interval, TC: total cholesterol, TG: triglycerides, PD: Parkinson’s disease, NOS: Newcastle-Ottawa Scale, US: the United States of America, NA: not available, BMI: Body Mass Index, M: male, F: female.

**Supplementary Table 3** Summarized dose-response data for the relationship between serum TC and PD risk.

| **Serum TC(mg/dL)** | **No. of cases** | **No. of participants or person-years/controls** | **Relative Risk(95% CI)** |
| --- | --- | --- | --- |
| **Rozani(2018) Male** |  | Person-years | HR |
| <180 | 175‡ | 294565§ | 1 |
| 180–209 | 144‡ | 294564§ | 0.82 (0.66–1.01) |
| ≥210 | 124‡ | 294564§ | 0.71 (0.55–0.93) |
| **Rozani(2018) Female** |  | Person-years | HR |
| <180 | 103‡ | 294565§ | 1 |
| 180–209 | 103‡ | 294564§ | 1.00 (0.74–1.34) |
| ≥210 | 97‡ | 294564§ | 0.93 (0.63–1.38) |
| **Hu(2008)** |  | Person-years | HR |
| ＜193 | 39 | 152754 | 1 |
| 193–228 | 141 | 272129 | 1.42 (1.00–2.03) |
| 232–266 | 195 | 263672 | 1.56 (1.10–2.21) |
| ≥270 | 250 | 232261 | 1.86 (1.31–2.63) |
| **Simon(2007) Male** |  | Person-years | RR |
| ＜159 | 27 | 38238 | 1 |
| 159–179 | 20 | 42245 | 0.64 (0.36–1.15) |
| 179–199 | 35 | 60639 | 0.78 (0.47–1.29) |
| 199–219 | 33 | 63238 | 0.69 (0.42–1.15) |
| 219–269 | 50 | 64281 | 1.02 (0.64–1.63) |
| ≥270 | 8 | 27423 | 0.36 (0.17–0.80) |
| **Simon(2007) Female** |  | Person-years | RR |
| ＜159 | 13 | 85109 | 1 |
| 159–179 | 17 | 82313 | 1.38 (0.67–2.85) |
| 179–199 | 28 | 107924 | 1.58 (0.82–3.04) |
| 199–219 | 26 | 144698 | 0.96 (0.49–1.86) |
| 219–269 | 49 | 188113 | 1.24 (0.67–2.28) |
| ≥270 | 8 | 64266 | 0.54 (0.22–1.30) |
| **de Lau(2006)** |  | Participants | HR |
| <228 | 30‡ | 15178§ | 1 |
| 228–255 | 25‡ | 15177§ | 0.82（0.48–1.41） |
| 259–286 | 16‡ | 15177§ | 0.55（0.30–1.02） |
| >286 | 16‡ | 15177§ | 0.55（0.30–1.04） |
| **Jeong(2019）** |  | Participants | HR |
| 160–200* | 606‡ | 32608 | 1 |
| 200–240* | 470‡ | 25310 | 1.05（0.87–1.28） |
| 240–300* | 156‡ | 8400 | 1.06（0.83–1.36） |
| ≥300* | 12‡ | 618 | 1.61（0.81–3.20） |

*: reference values were converted to the lowest exposure category form(see method).

‡: case number calculated from the reported number of total cases, number of participants or person-years for each quantile and RR(see method).

§: the number of participants/person-years for each quantile was assumed to be approximately equal(see method).

HR: hazard ratio, CI: confidence interval, TC: total cholesterol, PD: Parkinson’s disease.

**Supplementary Table 4** Summarized dose-response data for the relationship between serum LDL-C and PD risk.

| **Serum LDL-C(mg/dL)** | **No. of cases** | **No. of participants or person-years/controls** | **Relative Risk(95% CI)** |
| --- | --- | --- | --- |
| **Rozani(2018) Male** |  | Person-years | HR |
| <110 | 176‡ | 294565§ | 1 |
| 110–139 | 141‡ | 294564§ | 0.80 (0.65–0.98) |
| ≥140 | 126‡ | 294564§ | 0.72 (0.54–0.95) |
| **Rozani(2018) Female** |  | Person-years | HR |
| <110 | 106‡ | 294565§ | 1 |
| 110–139 | 104‡ | 294564§ | 0.98 (0.76–1.28) |
| ≥140 | 93‡ | 294564§ | 0.88 (0.62–1.29) |
| **Friedman(2013)** |  | Participants | OR |
| ＜100 | 137‡ | 15760 | 1 |
| 100–130 | 289‡ | 30189 | 1.10(0.90–1.32) |
| 130–160 | 267‡ | 26773 | 1.15(0.94–1.40) |
| ＞160 | 131‡ | 15247 | 0.99(0.77–1.27) |
| **Huang(2008)** |  | Participants | RR |
| <80 | 9 | 513 | 1 |
| 80–100 | 11 | 667 | 0.94(0.52–1.70)¶ |
| 100–120 | 8 | 837 | 0.54(0.29–1.03)¶ |
| 120–140 | 9 | 689 | 0.74(0.40–1.39)¶ |
| 140–160 | 3 | 325 | 0.53(0.22–1.26)¶ |
| ＞160 | 1 | 202 | 0.28(0.07–1.12)¶ |
| **Benn(2017)** |  | Participants | HR |
| ＜70* | 20 | 3774 | 1 |
| 70–100* | 75 | 18395 | 0.80(0.48–1.34) |
| 100–154* | 231 | 51957 | 0.69(0.43–1.12) |
| ≥154* | 113 | 26359 | 0.59(0.36–0.97) |

*: reference values were converted to the lowest exposure category form (see method).

‡: case number calculated from the reported number of total cases, number of participants or person-years for each quantile and RR(see method).

¶: RR and its 95% CI estimated from the number of cases and participants for each quantile (see method).

§: the number of participants/person-years for each quantile was assumed to be approximately equal(see method).

OR: odds ratio, RR: relative risk, HR: hazard ratio, CI: confidence interval, LDL-C: low-density lipoprotein cholesterol, PD: Parkinson’s disease.

**Supplementary Table 5** Subgroup analyses (serum HDL-C levels and PD risk).

| **Variables** | **No. of studies** | **Test of association** | | **Test of heterogeneity** | |
| --- | --- | --- | --- | --- | --- |
|  |  | **Pooled RR (95%CI)** | ***P* value** | **I^2^ (%)** | ***P* value** |
| **Study location** |  | | | | |
| Europe | 3 | 1.19(0.85–1.66) | 0.306 | 24.7 | 0.265 |
| Americas | 1 | 0.64(0.30–1.38) | 0.252 | — | — |
| Asia | 1 | 0.81 (0.80–0.83) | 0.000 | — | — |
| **Statin adjustment**  **/excluding statin users** |  | | | | |
| YES | 2 | 0.96(0.60–1.52) | 0.850 | 44.9 | 0.178 |
| NO | 3 | 1.03(0.58–1.85) | 0.913 | 67.6 | 0.046 |
| **Gender** |  | | | | |
| Male | 2 | 1.08(0.86–1.36) | 0.523 | 0 | 0.581 |
| Female | 2 | 1.62(0.76–3.45) | 0.209 | 63.0 | 0.100 |

RR: relative risk, CI: confidence interval, NOS: Newcastle-Ottawa Scale, PD: Parkinson’s disease, HDL-C: high-density lipoprotein cholesterol. *P* value for heterogeneity within subgroup.
