# Supplementary material for: Effects of Higher Serum Lipid Levels on the Risk of Parkinson's Disease: A Systematic Review and Meta-Analysis
Source: Front Neurol. 2020 Jun 26;11:597. doi: 10.3389/fneur.2020.00597 (PMC7332704; doi:10.3389/fneur.2020.00597)
Supplement: Supplementary file 2 [file Data_Sheet_1.pdf]

Supplementary Table 1 MEDLINE, EMBASE, ACP Journal Club, COCHRANE, and so on via OvidSP.

1. exp Parkinson Disease/
2. exp parkinsonism/
3. Parkinson$.ab,ti.
4. (PD or IPD).ab,ti.
5. (paralysis adj2 agitans).ab,ti.
6. or/1-5
7. exp cholesterol/
8. (cholesterol$ or epicholesterol$ or azacosterol$ or diazacholesterol$ or hydroxycholesterol$ or 19-iodocholesterol$ or iodocholesterol$ or ketocholesterol$ or oxocholesterol$ or HDL or LDL or HDL-C or LDL-C or lipoprotein$ or triglyceride$ or hypercholester$ or hyperlipoprotein$ or hyperlipidemi$ or hypertriglyceridemi$ or hypocholester$ or hypolipoprotein$ or hypolipidemi$ or hypotriglyceridemi$).ab,ti.
9. or/7-8
10. 6 and 9
11. exp animals/ not humans.sh.
12. 10 not 11
13. remove duplicates from 12
14. limit 13 to yr="1988-current"

Specific Electronic Databases with Dates of Coverage:
EBM Reviews - Cochrane Database of Systematic Reviews (2005 to March 19, 2020),
EBM Reviews - ACP Journal Club (1991 to February 2020),
EBM Reviews - Database of Abstracts of Reviews of Effects (1st Quarter 2016),
EBM Reviews - Cochrane Clinical Answers (March 2020),
EBM Reviews - Cochrane Central Register of Controlled Trials (February 2020),
EBM Reviews - Cochrane Methodology Register (3rd Quarter 2012),
EBM Reviews - Health Technology Assessment (4th Quarter 2016),
EBM Reviews - NHS Economic Evaluation Database (1st Quarter 2016),
Embase (1974 to 2020 March 25),
Ovid MEDLINE(R) and Epub Ahead of Print, In-Process & Other Non-Indexed Citations, Daily and Versions(R) (March 25, 2020)

Supplementary Table 2A Characteristics of included cohort studies.

Author (Year)	Study location	Cohort Name	Serum lipid parameters	Data	Follow-up time(years)	Cohort size (% M)	Age(years)	Cases	Outcome definition	Exposure	Quantity	RR(95% CI)	Variables adjusted	NOS
Rozani(2018)†	Israel/Europe	Maccabi Health Services	TC, LDL-C, HDL-C	Dose-response + High vs. Low	7.9 (mean)	261638 (42.7)	M 47.1(mean)<br>F 45.6(mean)	764	HR	TC<br><br>LDL-C<br><br>HDL-C	M tertiles:3 vs. 1<br>F tertiles:3 vs. 1<br>M tertiles:3 vs. 1<br>F tertiles:3 vs. 1<br>M tertiles:3 vs. 1<br>F tertiles:3 vs. 1	0.71(0.55–0.93)<br>0.93(0.63–1.38)<br>0.72(0.54–0.95)<br>0.88(0.62–1.29)<br>1.06(0.83–1.34)<br>1.21(0.85–1.74)	age, sex	9/9
Nam(2018)	Korea/Asia	National Health Insurance Service of South Korea	HDL-C, TG	High vs. Low	5.3 (mean)	17163560 (48.8)	≥40	44205	HR	HDL-C (mg/dL)<br>TG (mg/dL)	M ≥40 vs. ＜40*<br>F≥50 vs. ＜50*<br>⩾150 vs. ＜150	0.81(0.80–0.83)<br><br>1.13(1.10–1.15)	age, sex, smoking, alcohol, physical activity, income, BMI, eGFR, history of stroke	8/9
Huang(2015)	US/Americas	Atherosclerosis Risk in Communities Study	TC, LDL-C, HDL-C, TG	Dose-response + High vs. Low	21	15291 (44.9)	54.2(mean)	106	OR	TC<br>LDL-C<br>HDL-C<br>TG	Tertiles 3 vs. 1<br>Tertiles 3 vs. 1<br>Tertiles 3 vs. 1<br>Tertiles 3 vs. 1	0.43(0.22–0.87)<br>0.43(0.21–0.88)<br>0.64(0.30–1.38)<br>1.27(0.64–2.53)	age, sex, race, smoking, caffeine intake, statin	8/9
Hu(2008)	Finland/Europe	Monitoring Trends and Determinants of Cardiovascular Disease protocol	TC	Dose-response + High vs. Low	18.1 (mean)	50926 (48.6)	25–74	625	HR	TC (mg/dL)	Both ⩾270 vs. ＜193<br>M ⩾270 vs. ＜193<br>F ⩾270 vs. ＜193	1.86(1.31–2.63)<br>1.84(1.14–2.95)<br>1.86(1.11–3.13)	age, study year, BMI, SBP, education, leisure-time physical activity, smoking, alcohol, coffee, tea, cholesterol-lowering agent use, diabetes	8/9
Friedman(2013)	Israel/Europe	Health services clinical database	LDL-C	Dose-response + High vs. Low	7	94308 (47.2)	> 45	1035	OR	LDL-C (mg/dL)	＞160 vs. ＜100	0.99(0.77–1.27)	sex, low socioeconomic status, diabetes, ischemic heart disease, hypertension, cerebrovascular accidents, smoking, statin	8/9
Simon(2007)	US/Americas	Nurses' Health Study(NHS), Health Professionals Follow-up Study(HPFS)	TC	Dose-response + High vs. Low	NHS 12.6 (mean)<br>HPFS 18.1 (mean)	171879 (29.6)	M 40–75<br>F 30–55	530	RR	TC (high cholesterol)	Both yes vs. no<br>M yes vs. no<br>F yes vs. no	0.98 (0.82–1.19)<br>0.36(0.17–0.80)<br>0.54 (0.22–1.30)	age, smoking	6/9
Saaksjarvi(2015)	Finland/Europe	Mini-Finland Health Survey	TC, HDL-C, TG	High vs. Low	30	6641 (46.7)	30–79	89	RR	TC (mg/dL)<br>HDL-C (mg/dL)<br>TG (mg/dL)	⩾193 vs. ＜193<br>M ⩾39 vs. ＜39<br>F ⩾50 vs. ＜50<br>⩾150 vs. ＜150	0.85 (0.31–2.33)<br>0.74(0.22–2.43)<br><br>0.52(0.29–0.90)	age, sex, education, smoking, alcohol, exercise, serum vitamin D, coffee<br>age, sex, education, smoking, alcohol, exercise, serum vitamin D, coffee, BMI, blood pressure, TG or HDL-C, FBG	8/9

To be continued

Huang(2008)	Japan/Asia	Honolulu-Asia Aging Study	LDL-C	Dose-response + High vs. Low	3.3 (mean)	3233 (100)	77(mean)	41	RR	LDL-C (mg/dL)	⩾135 vs. ＜85	0.60(0.40–1.10)	age, smoking, coffee intake, bowel movement frequency, HDL-C, alcohol intake, presence of Apo e2 alleles, CASI	7/9
Grandinetti(1994)†	Japan/Asia	Honolulu-Asia Aging Study	TC	High vs. Low	26	8006 (100)	71–93	58	RR	TC (high cholesterol)	Yes vs. no	0.73(0.43–1.24)	age	6/9
De Lau(2006)	Netherland/Europe	Rotterdam Study	TC, HDL-C	Dose-response + High vs. Low	9.4 (mean)	6465 (41.1)	69(mean)	87	HR	TC (mg/dL)<br>HDL-C (mg/dL)	Both ＞286 vs. ＜228<br>M ＞274 vs. ＜216<br>F ＞293 vs. ＜236<br>Both ＞62 vs. ＜43<br>M ＞54 vs. ＜39<br>F ＞66 vs. ＜46	0.55(0.30–1.04)<br>0.86(0.36–2.02)<br>0.16(0.05–0.55)<br>1.81(0.96–3.42)<br>1.38(0.56–3.43)<br>2.69(1.11–6.49)	age, sex	8/9
Benn(2017) †	Denmark/Europe	Copenhagen General Population Study, Copenhagen City Heart Study	LDL-C	Dose-response + High vs. Low	8.2 (median)	111194 (45)	56(median)	460	HR	LDL-C (mg/dL)	⩾154 vs. ＜70*	0.59(0.36–0.97)	age, sex, birth year, smoking, alcohol, physical inactivity, income, education, menopause for women	8/9
Jeong(2019)†	Korea/Asia	National Health Screening Program	TC	Dose-response + High vs. Low	9.9(median)	76043 (51.5)	68.5(mean)	1427	HR	TC (mg/dL)	≥300 vs. 200–240	1.53(0.77–3.05)	age, sex, BMI, income, smoking, alcohol, physical activity, hypertension, diabetes, SBP, FBG	9/9

*Reference values were converted to the lowest exposure category form(see method). †: excluding statin users in the high vs. low analysis for serum lipid parameters. OR: odds ratio, RR: relative risk, HR: hazard ratio, CI: confidence interval, TC: total cholesterol, LDL-C: low-density lipoprotein cholesterol, HDL-C: high-density lipoprotein cholesterol, TG: triglycerides, PD: Parkinson's disease, NOS: Newcastle-Ottawa Scale, US: the United States of America. NA: not available, BMI: Body Mass Index, CASI: Cognitive Abilities Screening Instrument, SBP: systolic blood pressure, FBG: fasting blood glucose,eGFR: estimated glomerular filtration rate, M: Male, F: female.

Supplementary Table 2B Characteristics of included case-control studies.

Author (Year)	Study location	Serum lipid parameters	Data	Cases (% male)	Controls (% male)	Age	Outcome definition	Exposure	Quantity	RR(95% CI)	Variables adjusted	NOS
Vikdahl(2015)	Sweden/Europe	TC, TG	High vs. Low	84(54.7)	336(54.7)	NA	HR	TC (mg/dL)	Both Per 38.6mg/dL increase<br>M Per 38.6mg/dL increase<br>F Per 38.6mg/dL increase	0.95(0.77–1.19)<br>0.91(0.68–1.23)<br>0.95(0.68–1.33)	age, BMI, physical activity, smoking	8/9
								TG (mg/dL)	Per 88.5mg/dL increase	0.64(0.41–1.01)		
Miyake(2010)	Japan /Asia	TC	High vs. Low	249(37.4)	368(38.3)	Cases 68.5(mean)<br>Controls 66.6(mean)	OR	TC (high cholesterol)	Yes vs. no	0.58(0.33–0.97)	sex, age, region of residence, smoking, education, leisure-time exercise, BMI, dietary energy, cholesterol, vitamin E, alcohol, coffee, DGI	5/9
Savica(2012)†	US/Americas	TC	High vs. Low	196(61.7)	196(61.7)	Cases 71(median)<br>Controls NA	OR	TC (mg/dL)	＞300 vs. ＜300	1.49(0.73–3.04)	age, sex, smoking, coffee	7/9

†: excluding statin users in the high vs. low meta-analysis for serum lipid parameters. OR: odds ratio, RR: relative risk, HR: hazard ratio, CI: confidence interval, TC: total cholesterol, TG: triglycerides, PD: Parkinson's disease, NOS: Newcastle-Ottawa Scale, US: the United States of America, NA: not available, BMI: Body Mass Index, M: male, F: female.

Supplementary Table 3 Summarized dose-response data for the relationship between serum TC and PD risk.

Serum TC(mg/dL)	No. of cases	No. of participants or person-years/controls	Relative Risk(95% CI)
**Rozani(2018) Male**		Person-years	HR
<180	175‡	294565§	1
180–209	144‡	294564§	0.82 (0.66–1.01)
⩾210	124‡	294564§	0.71 (0.55–0.93)
**Rozani(2018) Female**		Person-years	HR
<180	103‡	294565§	1
180–209	103‡	294564§	1.00 (0.74–1.34)
⩾210	97‡	294564§	0.93 (0.63–1.38)
**Hu(2008)**		Person-years	HR
＜193	39	152754	1
193–228	141	272129	1.42 (1.00–2.03)
232–266	195	263672	1.56 (1.10–2.21)
⩾270	250	232261	1.86 (1.31–2.63)
**Simon(2007) Male**		Person-years	RR
＜159	27	38238	1
159–179	20	42245	0.64 (0.36–1.15)
179–199	35	60639	0.78 (0.47–1.29)
199–219	33	63238	0.69 (0.42–1.15)
219–269	50	64281	1.02 (0.64–1.63)
⩾270	8	27423	0.36 (0.17–0.80)
**Simon(2007) Female**		Person-years	RR
＜159	13	85109	1
159–179	17	82313	1.38 (0.67–2.85)
179–199	28	107924	1.58 (0.82–3.04)
199–219	26	144698	0.96 (0.49–1.86)
219–269	49	188113	1.24 (0.67–2.28)
⩾270	8	64266	0.54 (0.22–1.30)
**de Lau(2006)**		Participants	HR
<228	30‡	15178§	1
228–255	25‡	15177§	0.82（0.48–1.41）
259–286	16‡	15177§	0.55（0.30–1.02）
>286	16‡	15177§	0.55（0.30–1.04）
**Jeong(2019）**		Participants	HR
160–200*	606‡	32608	1
200–240*	470‡	25310	1.05（0.87–1.28）
240–300*	156‡	8400	1.06（0.83–1.36）
⩾300*	12‡	618	1.61（0.81–3.20）

*: reference values were converted to the lowest exposure category form(see method).
‡: case number calculated from the reported number of total cases, number of participants or person-years for each quantile and RR(see method).
§: the number of participants/person-years for each quantile was assumed to be approximately equal(see method).
HR: hazard ratio, CI: confidence interval, TC: total cholesterol, PD: Parkinson’s disease.

Supplementary Table 4 Summarized dose-response data for the relationship between serum LDL-C and PD risk.

Serum LDL-C(mg/dL)	No. of cases	No. of participants or person-years/controls	Relative Risk(95% CI)
**Rozani(2018) Male**		Person-years	HR
<110	176‡	294565§	1
110–139	141‡	294564§	0.80 (0.65–0.98)
⩾140	126‡	294564§	0.72 (0.54–0.95)
**Rozani(2018) Female**		Person-years	HR
<110	106‡	294565§	1
110–139	104‡	294564§	0.98 (0.76–1.28)
⩾140	93‡	294564§	0.88 (0.62–1.29)
**Friedman(2013)**		Participants	OR
＜100	137‡	15760	1
100–130	289‡	30189	1.10(0.90–1.32)
130–160	267‡	26773	1.15(0.94–1.40)
＞160	131‡	15247	0.99(0.77–1.27)
**Huang(2008)**		Participants	RR
<80	9	513	1
80–100	11	667	0.94(0.52–1.70)¶
100–120	8	837	0.54(0.29–1.03)¶
120–140	9	689	0.74(0.40–1.39)¶
140–160	3	325	0.53(0.22–1.26)¶
＞160	1	202	0.28(0.07–1.12)¶
**Benn(2017)**		Participants	HR
＜70*	20	3774	1
70–100*	75	18395	0.80(0.48–1.34)
100–154*	231	51957	0.69(0.43–1.12)
⩾154*	113	26359	0.59(0.36–0.97)

*: reference values were converted to the lowest exposure category form (see method).
‡: case number calculated from the reported number of total cases, number of participants or person-years for each quantile and RR(see method).
¶: RR and its 95% CI estimated from the number of cases and participants for each quantile (see method).
§: the number of participants/person-years for each quantile was assumed to be approximately equal(see method).
OR: odds ratio, RR: relative risk, HR: hazard ratio, CI: confidence interval, LDL-C: low-density lipoprotein cholesterol, PD: Parkinson’s disease.

Supplementary Table 5 Subgroup analyses (serum HDL-C levels and PD risk).

Variables	No. of studies	Test of association		Test of heterogeneity	
		Pooled RR (95%CI)	*P* value	I^2 (%)	*P* value
**Study location**					
Europe	3	1.19(0.85–1.66)	0.306	24.7	0.265
Americas	1	0.64(0.30–1.38)	0.252	—	—
Asia	1	0.81 (0.80–0.83)	0.000	—	—
**Statin adjustment /excluding statin users**					
YES	2	0.96(0.60–1.52)	0.850	44.9	0.178
NO	3	1.03(0.58–1.85)	0.913	67.6	0.046
**Gender**					
Male	2	1.08(0.86–1.36)	0.523	0	0.581
Female	2	1.62(0.76–3.45)	0.209	63.0	0.100

RR: relative risk, CI: confidence interval, NOS: Newcastle-Ottawa Scale, PD: Parkinson's disease, HDL-C: high-density lipoprotein cholesterol. *P* value for heterogeneity within subgroup.
